# An online gathering about the latest on molecular membrane biology

**DOI:** 10.1016/j.jbc.2021.101237

**Published:** 2021-09-24

**Authors:** Francesca Bottanelli, Anne Spang, Chris Stefan, Christian Ungermann

**Affiliations:** 1 Institute of Chemistry and Biochemistry, Freie Universität Berlin, Berlin, Germany; 2 Bioczentrum, University of Basel, Basel, Switzerland; 3 MRC Laboratory for Molecular Cell Biology, University College London, London, United Kingdom; 4 Department of Biology/Chemistry, Biochemistry, Osnabrück University, Osnabrück, Germany; 5 Center of Cellular Nanoanalytics (CellNanOs), Osnabrück University, Osnabrück, Germany

Gordon Research Conferences (GRC) are among the meetings that are particularly important as they are rather small and foster scientific exchange. Many established researchers participate and meet for a week, often in some remote location. The 2021 molecular membrane biology Gordon conference, usually held at Proctor Academy, was one of the many meetings affected when the SARS-CoV2 pandemic resulted in an immediate shutdown of all public life and world travel. Scientists and scientific organizations reacted immediately and discovered the advantages of online meetings. Several members of the membrane biology community felt that a complete halt of scientific conferences would particularly harm young investigators in their career path as they would not get the chance to present their data in front of the scientific community. As a result, we decided to take the initiative and try to find a format for an online meeting in 2021. Before starting, we sent around a survey to ask for interest and received overwhelming enthusiasm and support—a testament to the vitality of the membrane trafficking community.

We agreed on a format that would mimic somehow the in-person meeting: poster sessions, followed by a dense set of talks with sufficient time for discussion, a meet-the-speaker slot, then another set of talks, and eventually another poster session or meeting time. Talks were presented mostly by early career researchers, postdocs, or assistant professors. To foster discussion and presentation of unpublished data, talks were live and not recorded. Gather.town was used as a platform for poster sessions and social interactions. Very importantly, the meeting was completely free as funding was secured *via* four journals (Journal of Cell Biology, Journal of Cell Science, FEBS Journal, and Traffic Journal) and three German DFG-funded research consortia (SFB 944, 958, and TR 186), which covered the user fees for all participants.

## A virtual meeting success story

How did it work? Maybe we start from the end. “This was the best online meeting I attended during the entire pandemic period” was one of the enthusiastic responses. Why? Because the social platform was as close as one can get to real-life social interactions, and the sessions worked almost perfectly for our needs. Two poster sessions with a total of some 50 posters flanked the talks on each day. Posters were accessible, presenters available, and it felt like being in a real meeting. Poster sessions were well attended and so busy that people had to wait, just like an in-person meeting. But since posters were available throughout the entire meeting, people were also able to view posters in their own spare time. The entire community was present, from PhD students to postdocs, young investigators, and established figures in the field. For the talks, we explicitly asked speakers to keep to their time to 10 min to allow for questions, asked the audience to write their questions into the chat and the chair to act as a mediator between questions and speaker. This made sessions dense, but also to the point. As all speakers kept to their time, and the chairs monitored their sessions so well, enough time was left for questions. We were impressed, how much science can be compressed in a short talk, and how excellent all speakers presented. The more exciting the talk, the earlier the questions appeared in the chat. As the chairs combined questions and condensed the information, the most important open issues were immediately addressed. Breaks were reserved to meet-the-speaker, and by the end of the day—or better the end of the meeting—many of us realized how long they had been sitting fixed in front of their screen without moving much. Throughout the meeting, 200–250 people were listening to the talks, more than 150 were present at the poster sessions. It was as good as it gets.

## The science

We selected speakers based on their science and tried to balance the field and explicitly focused on postdocs and early career investigators. As the science of the talks was meant to be “off the record” and live, in the spirit of a Gordon Research Conference, we decided to just let the program speak for itself (see supporting information). **Liz Miller** (MRC Cambridge, United Kingdom) opened the dances of the first ER-focused session with a talk about the role of unstructured regions in assembly of the COPII coat (unpublished and (1)). The session featured structural work by **Tino Pleiner** (Caltech, USA) and **Antonio Galindo** (MRC, United Kingdom). Tino's work shed light on the mechanisms of membrane protein insertion into the ER and the regulation of the human 9-subunit membrane protein insertase ER membrane protein complex (EMC) *via* the kinase WNK1 (2, 3). Antonio's TRAPPIII structure reveals insights into the mechanisms of Rab1 activation to regulate secretory trafficking (4). **Ishier Raote** (CRG, Spain) shared the latest about the function of TANGO1 in creating a tunnel at the ER for export of bulky cargo (5, 6, 7), including some exciting unpublished results about the role of liquid–liquid phase separation (LLPS) in the assembly of TANGO1 at ER exit sites. Sessions 2 and 3, focused on trafficking through the Golgi compartments and secretory pathway, included talks by **Ivan Castello Serrano** (University of Virginia, USA) about the importance of lipid rafts for export of transmembrane proteins from the Golgi network, and **Anup Parchure** (Yale University, USA) whose work suggests that secretory granule biogenesis at the TGN is facilitated by LLPS. **Lauren Jackson** (Vanderbilt University, USA) presented structural work shedding light on the regulation of post-Golgi cargo recycling in yeast *via* the ArfGAP Glo3 (8). **Mara Duncan** (University of Michigan, USA) presented some recent work from her lab where a human iPSC model system combined with proximity-based proteomics was used to identify trafficking machinery responsible for polarized trafficking (9). **David Murray** (University of Dundee, United Kingdom) presented exciting unpublished data about the functional characterization of the exocyst tethering complex and its cross talk with phosphoinositide signaling. The session was closed by **Allison Zajac** (University of Chicago, USA), whose latest preprint (10) highlights the role of Kinesin-3 and -1 motors in basolateral secretion in epithelial cells. The first day wrapped up with a session focused on mitochondria/peroxisomal membranes packed with excellent unpublished findings. **Tim König** (McGill University, Canada)'s work reveals the lipid and protein composition and the mechanisms of formation of mitochondrial-derived vesicles (MDVs). **Joshua Pemberton** (NICHD, USA) developed tools to alter the lipid composition of the outer mitochondrial membrane and showed that localized production of diacylglycerol altered mitochondrial morphology. **Cansu Kuey** (University of Warwick, United Kingdom) showed that acute redistribution of clathrin mediated endocytosis machinery on mitochondria is sufficient to drive the formation of clathrin-coated vesicles. **Noa Dahan** (Weizman Institute, Israel) talked about the importance of local mRNA translation for peroxisomal function.

The second day kicked off with a session on the endocytic pathway, in which **Andreas Mayer** (University of Lausanne, Switzerland) discussed his unpublished data on the regulation of membrane fission in the endolysosomal system by CROP, a novel complex consisting of Atg18 and the retromer complex. Staying on the membrane fission theme, **Sho Suzuki** (Cornell University, USA) revealed how the yeast Mvp1/SNX8 protein and the dynamin-like GTPase Vps1 drive fission and endosomal recycling. While **Doris Höglinger** (Biochemistry Center Heidelberg, Germany) discussed routes for cholesterol and sphingolipid transport, **Florian Fröhlich** (University of Osnabrück, Germany) presented his proteomic mapping of the endolysosomal system, which is available as a preprint (11). In the second session of the day, **Kamalesh Kumari** (Weizmann Institute, Israel) argued that mechanochemical sequestration and vesicle crumpling drive exocytosis while maintaining apical membrane homeostasis. On the theme of membrane compartmentalization, **Kasey Day** (University of Texas, USA) provided evidence that liquid-like protein droplets are important for endocytic vesicle formation, and **Claudia Matthaeus** (NIH, USA) discussed the role of lipid uptake in caveolae. **Henning Arlt** (Harvard University, USA) provided structural and mechanistic insights into lipid droplet biogenesis. **Andrés Guillén-Samander** (Yale University, USA) revealed how VPS13D together with Miro facilitates interorganelle contacts between ER and mitochondria and peroxisomes (12). These presentations were followed by two talks on ESCRT-III: Jiwei Liu (Rosalind Franklin Institute, UK) provided compelling evidence for the presence of ESCRT-III membrane remodelers in bacteria, while **Joachim Moser von Filseck** (University of Geneva, Switzerland) provided molecular details on ESCRT-III membrane remodeling in eukaryotes (13). The last three talks of the day were on autophagy. **Tara Fisher** (NIH, USA) started with a presentation on how STING induces LC3 lipidation (14). **Rachel Ulferts** (Francs Crick Institute, United Kingdom) then discussed the role of the V-ATPase in inflammation and autophagy. Finally, **Florian Wilfling** (Max-Planck-Institute for Biophysics, Germany) shared his data on intrinsic autophagy receptors and how they function in the disposal of macromolecular machines including stalled endocytic machinery (15).

## The lessons

Virtual molecular membrane biology 2021 was a success mainly because junior scientists were given a platform to present their exciting (and unpublished!) research. Given that many talks at previous meetings were presented by established scientists, who are also one reason to attract others (and some would otherwise also not join the meeting if not invited), the online meeting could make sure that more junior researchers were allowed to present and make their presence to the scientific community.

In-person meetings mean frequent get-togethers on all occasions and time to talk and interact beyond listening to the talks. As scientists, we need this type of interaction to process what we learned and develop new ideas and directions. In an online format, travel, organization, and a week away from the lab and family are not happening, with all its pros and cons. Will such online meetings continue to work in the future? Or will we return to what we had before? We believe virtual gatherings cannot completely substitute for in-person meetings, but they can be a powerful way to keep the community connected outside of conference season. It definitely worked for us.

## Supporting information

This article contains supporting information.
