# Supplementary material for: An online gathering about the latest on molecular membrane biology
Source: J Biol Chem. 2021 Sep 24;297(5):101237. doi: 10.1016/j.jbc.2021.101237 (PMC8605330; doi:10.1016/j.jbc.2021.101237)
Supplement: Supporting information [file mmc1.pdf]

July 2021

Dear friends and colleagues,

2021 marks the beginning of the vaccination campaign, but unfortunately not the end of the pandemic. Many meetings were cancelled or postponed, including the Gordon Research Conference on “Molecular Membrane Biology”, which should happen in 2023.

After many emails, the four of us, Anne Spang, Francesca Bottanelli, Chris Stefan and Christian Ungermann, took the initiative to do ‘something’. We felt that the community is longing for a vivid exchange and therefore searched for an alternative possibility to give in particular the young investigators a chance to present their progress and become more visible. The facts speak for themselves, 452 people registered with 120 wanted to talk (a tough decision) - from PhD students to many senior faculty – we are absolutely flashed by the positive response.

What came out is this format, a three-day event of talks, posters and meeting time.

We will use the **gather.town platform** starting with a small orientation on July 18 at 5 pm (European summer time), which is adjusted to our needs - to meet and talk, visit posters and meet the speaker or talk to editors. You will find a small manual how to use it in the attachment. At the core, we will have **28 talks** by young (and a bit older) investigators and faculty. You find the access at the auditorium. The four sessions on each day are interrupted by breaks with the opportunity to meet with the speakers of the session within the poster room. You can use the platform (beach site, poster session, lawn) to meet (by tracing people or contacting them via the chat) and then interact.

Three **poster sessions** happen before and after the talks. We recommend that you look at the posters before the meeting and use the time at the posters to pose your questions.

Thanks to the **sponsoring**, the meeting is for free. We will have poster prizes (made possible to FEBS Journal and Traffic) that we will award after the last session.

We hope that you will take advantage of the meeting, meet many new colleagues, catch up with the ones you know and enjoy the exchange. We are aware that it would be all much better in-person but let us try to make the best out of this. The meeting will in the end be as good as you make it.

We look forward to your coming!

Francesca Bottanelli
Anne Spang
Chris Stefan
Christian Ungermann

Online Molecular Membrane Biology Conference 2021, July 18-20

Note - All times are European Central times

Sunday, 18th of July

5:00 –	**Try out Conference Website - Meet and Greet**

Monday, 19th of July

3:00 – 3:50	Social Mixer/**Poster Session 1**	**UK. 2 pm; Boston, 9 am, LA, 6 am, Tokio, 9 pm, Sydney, 10 pm**
3:50 – 4:00	Welcome (**Auditorium**)	Christian Ungermann, Francesca Bottanelli, Anne Spang, Chris Stefan

**Session 1 (Auditorium)**	**Chair: Felix Campelo**	**Support: Christian Ungermann**	**Meet-the-speaker at "tables" in poster area**
4:00 – 4:15	**Liz Miller** (MRC Cambridge)	Order from disorder: intrinsically disordered domains in COPII coat assembly	**1**
4:15 – 4:30	**Tino Pleiner** (Caltech, Pasadena, USA)	Structure and assembly of the human ER membrane protein complex.	**2**
4:30 – 4:45	**Ishier Raote** (CRG, Barcelona, Spain)	TANGO1 builds a multi-organelle machine for bulky cargo secretion	**3**
4:45 – 5:00	**Antonio Galindo** (MCR Cambridge, UK)	Falling into the TRAPP: A cryo-EM structure of TRAPPIII, the multisubunit complex that activates the GTPase Rab1	**4**
**Session 2**	**Chair: Chris Burd**	**Support: Anne Spang**	
5:00 – 5:15	**Ivan Castello Serrano** (University of Virginia, Charlottesville, USA)	Rafting in a RUSH: membrane microdomains in secretory trafficking	**5**
5:15 – 5:30	**Lauren Jackson** (Vanderbilt University, Nashville, USA)	An interaction between beta'-COP and its ArfGAP, Glo3, is required to maintain post-Golgi cargo recycling	**6**
5:30 – 5:45	**Anup Parchure** (Yale University, New Haven, USA)	Liquid liquid phase separation (LLPS) facilitates biogenesis of secretory storage granules	**7**
5:45 – 6:15	**Break / Meet the speaker** at **poster area**		
**Session 3**	**Chair: Gillian Griffiths**	**Support: Francesca Bottanelli**	
6:15 – 6:30	**Mara Duncan** (University of Michigan, Ann Arbor, USA)	Novel roles for AP-1 in morphogenesis revealed by proteomic analysis of pluripotent stem cells	**1**
6:30 – 6:45	**David Murray** (University of Dundee, UK)	A mechanism for exocyst-mediated cargo delivery in polarized trafficking	**2**
6:45 – 7:00	**Allison Zajac** (University of Chicago, USA)	Basement membrane protein secretion is directed to a basal region of the basolateral membrane in epithelial cells by kinesin-3 and kinesin-1 motors	**3**
**Session 4**	**Chair: Maya Schuldiner**	**Support: Chris Stefan**	
7:00 – 7:15	**Tim König** (McGill University, Montreal, Canada)	Mechanisms of MDV biogenesis: MIRO1/2 and DRP1 mediate removal of assembled protein complexes	**4**
7:15 – 7:30	**Joshua Pemberton** (NICHD, Bethesda, USA)	Acute manipulation of outer membrane phospholipid composition directly alters mitochondrial dynamics and ultrastructure.	**5**
7:30 – 7:45	**Noa Dahan** (Weizman Institute, Rehovot, Israel)	Peroxisomal mRNA localized translation supports optimal organelle function	**6**
7:45 – 8:00	**Cansu Kuey** (University of Warwick, UK)	Reconstituting clathrin-coated vesicle formation on mitochondria	**7**
8:00 – 8:30	**Meet the speaker** at **poster area**		
8:00 – 9:00	Social Mixer/**Poster Session 2**		

Tuesday, 20th of July

3:00 – 4:00	Social Mixer/**Poster Session 3**		
**Session 5 (Auditorium)**	**Chair: Tamas Balla**	**Support: Christian Ungermann**	
4:00 – 4:15	**Andreas Mayer** (University of Lausanne, Switzerland)	The CROP complex in membrane fission on endo-lysosomal compartments.	**1**
4:15 – 4:30	**Sho Suzuki** (Cornell University, Ithaca, USA)	A PX-BAR protein Mvp1/SNX8 and a dynamin-like GTPase Vps1 drive endosomal recycling	**2**
4:30 – 4:45	**Doris Höglinger** (Biochemistry Center Heidelberg, Germany)	Novel lysosome-targeted lipid probes reveal a common route for cholesterol and sphingolipid export	**3**
4:45 – 5:00	**Florian Fröhlich** (University of Osnabrück, Germany)	All roads lead to the lysosome - Proteomic mapping of endo-lysosomal trafficking in *S. cerevisiae*	**4**
**Session 6**	**Chair: Suzanne Pfeffer**	**Support: Francesca Bottanelli**	
5:00 – 5:15	**Kamalesh Kumari** (Weizmann Institute, Revohot, Israel)	During exocrine secretion, exocytosis by vesicle crumpling and mechanochemical sequestration maintains apical membrane homeostasis.	**5**
5:15 – 5:30	**Kasey Day** (Univerity of Texas, Austin, USA)	Liquid-like protein droplets catalyze assembly of endocytic membrane vesicles	**6**
5:30 – 5:45	**Claudia Matthaeus** (NIH, Bethesda, USA)	Caveolae mediated lipid uptake and trafficking in health and disease	**7**
5:45 – 6:15	**Break / Meet the speaker** at **poster area**		
**Session 7**	**Chair: Mike Henne**	**Support: Anne Spang**	
6:15 – 6:30	**Henning Arlt** (Harvard University, Boston, USA)	New mechanistic insights into lipid droplet biogenesis	**1**
6:30 – 6:45	**Andrés Guillén-Samander** (Yale University, New Haven, USA)	VPS13D bridges the ER to mitochondria and peroxisomes via Miro	**2**
6:45 – 7:00	**Jiwei Liu** (Rosalind Franklin Institute, UK)	Bacterial Vipp1 and PspA are members of the ancient ESCRT-III membrane-remodelling superfamily	**3**
**Session 8**	**Chair: Heidi McBride**	**Support: Chris Stefan**	
7:00 – 7:15	**Joachim Moser von Filseck** (University of Geneva, Switzerland)	Molecular details of ESCRT-III-mediated membrane remodelling	**4**
7:15 – 7:30	**Tara Fischer** (NIH, Bethesda, USA)	Behind the VAIL: STING, the V-ATPase, and autophagy?	**5**
7:30 – 7:45	**Rachel Ulferts** (Francis Crick Institute, London, UK)	The V-ATPase as the master controller of organelle homeostasis, inflammation and autophagy.	**6**
7:45 – 8:00	**Florian Wilfling** (Max-Planck Institute of Biophysics, Frankfurt, Germany)	Intrinsic autophagy receptors - a way to dispose of macromolecular machines	**7**
8:00-8:10	**Anne, Christian, Francesca, Chris**	Final words, Poster prizes	
8:10 – 8:40	**Meet the speaker** at **poster area**		
8:10 –	Social Mixer		
